# Successful Treatment of a 15-Year-Old Nonunion of a Midshaft Clavicle Fracture Causing Brachial Plexus Compression

**DOI:** 10.1155/2017/5105670

**Published:** 2017-09-10

**Authors:** Annemarijn Teunis, Rianne M. H. A. Huis In 't Veld, Vincent E. J. A. de Windt, Sjoerd van Raak, Anne J. H. Vochteloo

**Affiliations:** Centre for Orthopaedic Surgery, OCON, P.O. Box 546, 7555 DL Hengelo, Netherlands

## Abstract

A 49-year-old man with a 15-year-old nonunion of a midshaft clavicle fracture suffered from progressive tingling in his entire arm and fingers for two years, due to irritation of the brachial plexus in the costoclavicular space, especially upon elevation of the arm. After open reduction and internal plate fixation, all symptoms were resolved and complete consolidation of the fracture was achieved at one-year follow-up. This case demonstrates two things: brachial plexus compression can occur even many years after a nonunion of a clavicle fracture and union can be still achieved, even in a longstanding nonunion.

## 1. Introduction

Clavicle fractures account for 3–10% of all fractures, with an incidence of 30 per 100.000 inhabitants in adults [1, 2]. Fractures of the midshaft are the most common (over 69%) [2].

Nonunion after a midshaft fracture occurs in 5–20% of cases after conservative treatment [3]. A meta-analysis on operative versus conservative treatment of midshaft clavicle fractures reports a 14% incidence of brachial plexus involvement after conservative treatment [4]. Brachial plexus injury following clavicular fracture is a rare condition, occurring in less than 1% of cases [5]. A brachial plexus injury arising from callus formation following clavicular nonunion is even rarer. Acute brachial plexus injuries usually arise within a few weeks to months. However, there is one previously mentioned case which describes a delayed onset [6]. We add a further case of delayed onset of brachial plexus compression, 13 years after the injury.

We describe the successful treatment of a patient with progressive complaints of a delayed brachial plexus compression due to a 15-year-old nonunion midshaft clavicle fracture.

## 2. Case Report

A 49-year-old man presents with progressive complaints in his left arm over the past two years. He experiences a tingling and a numb feeling in his entire arm especially when walking or driving. These complaints initiate in the hand, digits two to four in particular, and progress proximally over time.

Furthermore, the patient has an annoying sensation of two rubbing clavicular fracture parts. Fifteen years ago he fell from a height of five meters during work. As a result of this accident he sustained a midshaft clavicle fracture on the left side, which was treated conservatively.

Physical examination revealed a drooping left shoulder, a visible thickening, and an abnormally shaped clavicle. Active and passive full range of motion of the shoulder was found.

Neurological examination showed symmetrical low reflexes and a normal sensibility. When raising the arm and keeping it elevated, tingling and numbness occurred.

**Figure 1:**
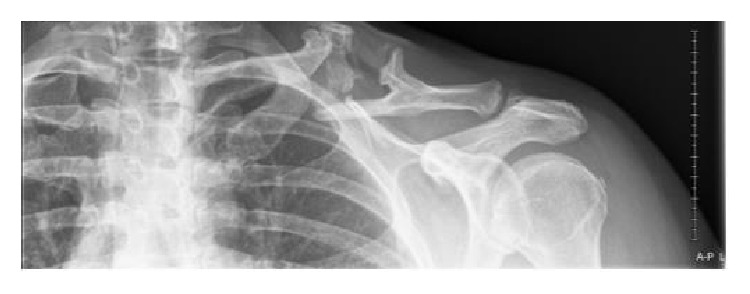
Before surgery.

X-rays showed a nonunion of a midshaft clavicle fracture with extension callus formation (Figure 1).

**Figure 2:**
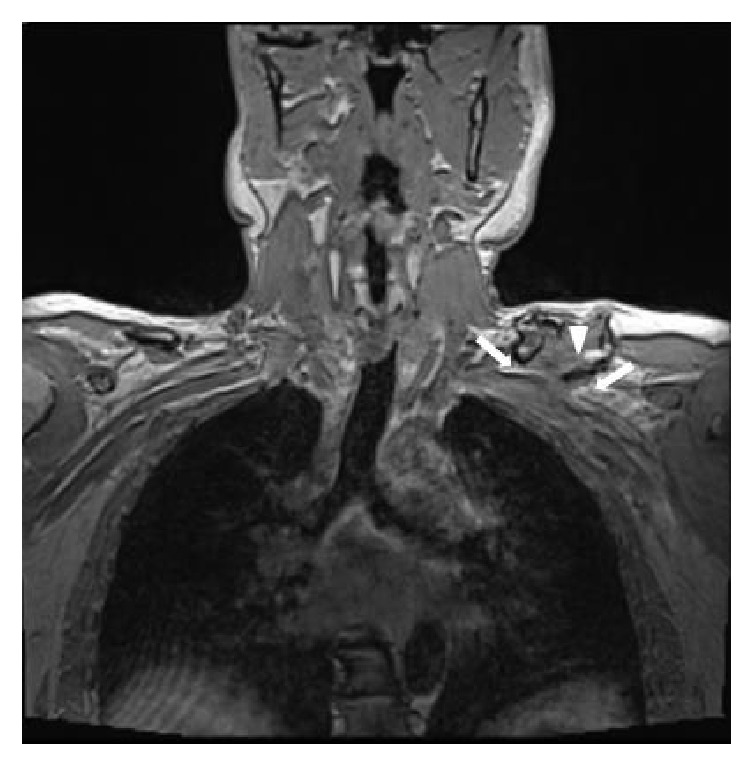
Coronal three-dimensional T1-weighted volumetric interpolated breath-hold image shows compression of the infraclavicular part of the brachial plexus (white arrow) by an inferior bone fragment (white arrowhead).

An MRI showed granulation tissue around the brachial plexus, without revealing an injury of the plexus itself (Figure 2).

**Figure 3:**
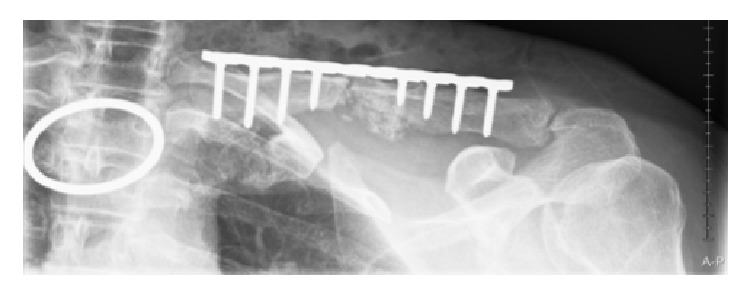
First day after implementation of plate osteosynthesis.

The diagnosis was therefore a hypertrophic nonunion of a midshaft clavicle fracture causing compression of the brachial plexus, without damage to the plexus. We decided to perform an open reduction and internal fixation of the clavicle, using a precontoured locking plate (Perilock, Smith & Nephew, Warsaw, USA) (Figure 3).

**Figure 4:**
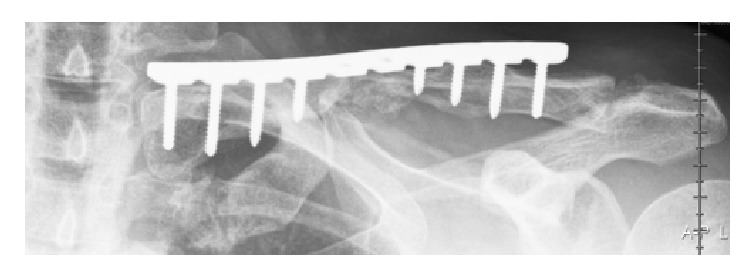
Six months after plate osteosynthesis. There is a minimal callus forming, without consolidation.

**Figure 5:**
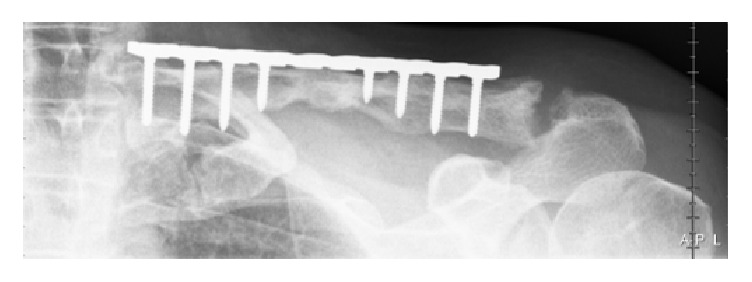
Twelve months after plate osteosynthesis. Good development of the slowed consolidation.

Intraoperatively, extensive preparation to free both ends of the clavicle from scar tissue was performed, both ends were trimmed to fit as well as possible, an autologous bone graft was performed (obtained from the resected parts), the medullary canal was opened, and the plate was fixed. At the first postoperative visit (six weeks) the tingling sensation had gone almost completely. Six months postoperatively, the patient reported no pain, and he had obtained a full range of motion. Despite these satisfactory results, complete healing of the fracture was not yet seen on the X-rays (Figure 4). However, during the final check-up, 12 months after treatment, a complete healing of the clavicula fracture was observed (Figure 5).

## 3. Discussion

We presented a patient with progressive compression of the brachial plexus which arose 13–15 years after a nonunion midshaft clavicle fracture, which was treated successfully with plate osteosynthesis of the clavicle. This case shows that even 15 years after a clavicle fracture, compression of the brachial plexus can still occur and union can be achieved with a nonunion repair (plate osteosynthesis and autologous bone graft).

Brachial plexus injury or compression may occur, both in the acute and in the chronic phases [7]. In the majority of cases, plexus neuropathy based on pressure may develop from three weeks to seven years after the damage [6, 7] (Table 1). Only one case with a delayed onset of 7–10 years is known; however, this case is dated from several decades ago (1949) [6]. Both our case and Campbell et al.'s [6] describe a favorable outcome with a complete recovery, despite the substantial delay. This shows that there is no reason not to operate when there is a considerable delay in the onset of brachial plexus complaints resulting from a nonunion clavicular fracture in the past.

Compression of the brachial plexus in patients who have not been operated on is reported to be 14% [4]. This circumstance is almost exclusively caused by hypertrophic bone growth with a nonunion midshaft clavicle fracture [7]. Herein compression of the brachial plexus takes place within the costoclavicular area, which is formed by the middle one-third of the clavicle and the first rib. Often there is persistent pressure on the root inferior trunk, medial fasciculus [16]. Patients may experience pain in the shoulder or armpit and a numb or tingling feeling in (parts of) the arm or hand [17]. In the current case the patient experienced tingling and numbness in the hand, radiating to the shoulder, without loss of function.

The prognosis of compression of the brachial plexus is generally poor; however, it does depend on the severity of the lesion. For acute plexus injuries, most improvement can be expected in the first six months after neurolysis [18]. Known cases do not provide a clear time scale during which the brachial plexus should be operated upon; however, based on neurobiology knowledge it is important to treat an injury as soon as possible [14].

In this case, the patient already showed signs of improvement within six weeks and recovered completely after six months. Based on the articles mentioned in Table 1, a positive outcome for a delayed onset can be expected (even after years).

To conclude, this case demonstrated a delayed brachial plexus compression due to a 15-year-old nonunion of a midshaft clavicle fracture, which resolved completely after open reduction and plate fixation.

## Figures and Tables

**Table 1:** Overview cases with brachial plexopathy resulting from a nonunion hypertrophic clavicle fracture.

	Date of publication	Study sample	(Delayed) time span/onset of symptoms	Treatment strategy	Laboratory data	Postoperative course
*Our case report*						

Teunis et al.		*N* = 1	*13 yrs*	surgical decompression + ORIF	*X-ray, MRI*	*Complete recovery after 1 year*

*Available literature (full text)*						

Berkheiser [8]	1937	*N* = 6	3 months to 1 year	Plexus brachial involvement arose after the first operation	X-ray	Full recovery after a second operation

Storen [9]	1946	*N* = 1	3 yrs	Resection + metal suture	X-ray	After 5 months a second operation, because of pain. New suture, after that full recovery in 14 months

Campbell et al. [6]	1949	*N* = 1	7 yrs	Removing of the callus and scar tissue	X-ray	Complete recovery after 4 weeks

Neviaser [10]	1963	*N* = 1	3 months	Resection of the middle part of the clavicle	X-ray	Complete recovery after 2 months

Miller and Boswick [11]	1969	*N* = 4	3, 4, 12, and 5 wk	Claviculectomy, exploration, ORIF, exploration	X-ray	Complete recovery in 1 year, unknown, 6 months, and 4 months

Wilkins and Johnston [12]	1983	*N* = 2	12 and 14 wk	Resection of 1/3 of clavicle, ORIF	Unknown	Pain when lying on the affected side, complete recovery

Kay and Eckardt [7]	1986	*N* = 1	3 wk	ORIF	X-ray + EMG	*Complete recovery after 10 months*

Connolly and Dehne [13]	1989	*N* = 4	8, 18, 8, and 4 months	Resection, resection, resection, pinning	X-ray	The patient with pinning technique recovered completely, the other cases unknown

Derham et al. [14]	2007	*N* = 1	8 wk	Surgical decompression + ORIF	X-ray, MRI, EMG	Complete recovery after 3 months

Thavarajah and Scadden [15]	2013	*N* = 1	Directly after first procedure (8 months)	ORIF first operation, surgical decompression second operation	X-ray	MRC^*∗*^ grade 4/5 power from C5–T1

^*∗*^British Medical Research Council (MRC).

## References

[1] Postacchini F., Gumina S., de Santis P., Albo F. (2002). Epidemiology of clavicle fractures. *Journal of Shoulder and Elbow Surgery*.

[2] Robinson C. M. (1998). Fractures of the clavicle in the adult. Epidemiology and classification. *Journal of Bone and Joint Surgery*.

[3] Murray I. R., Foster C. J., Eros A., Robinson C. M. (2013). Risk factors for nonunion after nonoperative treatment of displaced midshaft fractures of the clavicle. *Journal of Bone and Joint Surgery - Series A*.

[4] McKee R. C., Whelan D. B., Schemitsch E. H., McKee M. D. (2012). Operative versus nonoperative care of displaced midshaft clavicular fractures: a meta-analysis of randomized clinical trials. *The Journal of Bone & Joint Surgery—American Volume*.

[5] Rowe C. R. (1968). An atlas of anatomy and treatment of midclavicular fractures. Orthop Relat Res. *Clin Orthop Relat Res*.

[6] Campbell E., Howard W. P., Burklund C. W. (1949). Delayed brachial plexus palsy due to ununited fracture of the clavicle: Report of a case. *Journal of the American Medical Association*.

[7] Kay S. P., Eckardt J. J. (1986). Brachial plexus palsy secondary to clavicular nonunion. *Case Report and Literature Survey*.

[8] Berkheiser E. J. (1937). Old ununited clavicular fractures in the adult. *Surg Gynecol Obstet*.

[9] Storen H. (1946). Old clavicular pseudarthrosis with late with late appearing neuralgias and vasomotoric disturbances cured by operation. *Acta Chir Scand*.

[10] Neviaser J. S. (1963). The treatment of fractures of the clavicle. *Surgical Clinics of North America*.

[11] Miller D., Boswick J. (1969). Lesions of the brachial plexus associated with fracture of the clavicle. *Clin. Orthop*.

[12] Wilkins R. M., Johnston R. M. (1983). Ununited fractures of the clavicle.. *The Journal of Bone & Joint Surgery*.

[13] Connolly J. F., Dehne R. (1989). Nonunion of the Clavicle and Thoracic Outlet Syndrome. *The Journal of Trauma: Injury, Infection, and Critical Care*.

[14] Derham C., Varghese M., Deacon P., Spencer N., Curley P. (2007). Brachial plexus palsy secondary to clavicular nonunion. *Journal of Trauma - Injury, Infection and Critical Care*.

[15] Thavarajah D., Scadden J. (2013). Iatrogenic postoperative brachial plexus compression secondary to hypertrophic non-union of a clavicle fracture. *Annals of the Royal College of Surgeons of England*.

[16] Onstenk R., Malessy M. J. A., Nelissen R. G. H. H. (2001). Plexus-brachialisletsel door niet genezen of in afwijkende stand genezen claviculafracturen. *Ned Tijdschr Geneeskd*.

[17] Bromberg M. B., Shefner J. M., Dashe J. F. Brachial plexus syndromes. http://www.uptodate.com.proxy-ub.rug.nl/contents/brachial-plexus-syndromes?source=machineLearning&search=plexus+brachialis&selectedTitle=1~150&sectionRank=2&anchor=H8#H5.

[18] Midha R. (1997). Epidemiology of brachial plexus injuries in a multitrauma population. *Neurosurgery*.

